# Synthesis, characterization, DFT calculation, and biological activity of a new Schiff base ligand and its ZnO and Co_3_O_4_ nano-metal oxide complexes

**DOI:** 10.1186/s13065-025-01673-1

**Published:** 2025-12-12

**Authors:** Abeer W. Hasan, Zainab N. Zubaidi, Maha Y. Mustafa, Lekaa K. Abdul Karem, Riyadh M. Ahmed, Osama’a A. Y. Al-Samrai, Mouhaned Y. Al-Darwesh, Ibrahim Nazem Qader, Karukh Ali Babakr

**Affiliations:** 1 Ministry of Education, Directorate General of Education Karkh Ι, Baghdad, Iraq; 2 Institute of Medical Technology-Baghdad, Middle Technical University, Baghdad, Iraq; 3 Technical Nursing Department, Technical Institute Suwaira, Middle Technical University, Wasit, Iraq; 4 Department of Chemistry, College of Education for Pure Science Ibn Al-Haitham, University of Baghdad, Baghdad, Iraq; 5 Department of Chemistry, College of Education, University of Samarra, Samarra, Iraq; 6 Department of Chemistry, College of Sciences, University of Anbar, Anbar, Iraq; 7 Department of Physics, College of Science, University of Raparin, Rania, Sulaymaniyah, 46012 Iraq; 8 Chemistry Department, College of Science, University of Raparin, Rania, Sulaymaniyah, 46012 Iraq; 9 Department of Pharmacy, College of Pharmacy, Knowledge University, Erbil, 44001 Iraq

**Keywords:** Schiff-base ligand, DFT calculation, Co_3_O_4_ NPs, Biological activity

## Abstract

A novel heterocyclic Schiff base ligand, {2,2′-((4-chloro-1,3-phenylene)-bis(oxy))bis-(N′-((E)-(1 H-benzo[1-3]-triazole-1-yl)methylene)acetohydrazide)}, was synthesized and coordinated with Co(II) and Zn(II) chlorides to yield two metal complexes. The ligand and complexes were analyzed using FT-IR, ¹H and ¹³C NMR, UV-visible spectroscopy, and mass spectrometry, which provided spectral shifts typical of coordination involving the imine nitrogen and amide carbonyl groups. DFT calculations (B3LYP/LanL2DZ) showed that complexation decreased the energy gap between HOMO and LUMO from 4.26 eV (free ligand) to 3.18 eV and 2.66 eV for Zn(II) and Co(II) complexes, respectively, showing increased chemical reactivity. Similarly, the electrophilicity index increased to 33.81 eV for the Zn complex and 40.97 eV for the Co complex, indicating increased electron-accepting ability and potential biological activity. Thermal decomposition of Zn and Co complexes yielded ZnO NPs (mean crystallite size 35.6 nm) and Co_3_O_4_ NPs (33.8 nm), as evidenced by powder X-ray diffraction (XRD) and transmission electron microscopy (TEM). Antibacterial activity against Gram-positive bacteria (*Staphylococcus aureus*,* Bacillus subtilis*) and Gram-negative bacteria (*Escherichia coli*,* Pseudomonas aeruginosa*) was found to show that ZnO NPs had the largest inhibition zones (up to 28 mm), followed by metal complexes [Zn(L)]Cl₂ and [Co(L)]Cl_2_, whereas the free ligand had very poor activity (only inhibition zones of 11–13 mm).

**Supplementary Information:**

The online version contains supplementary material available at 10.1186/s13065-025-01673-1.

## Introduction

A wide variety of chemical compounds containing nitrogen and oxygen donor atoms play crucial roles in multiple disciplines, including analytical chemistry, biology, industrial applications, and agriculture [1, 2]. Schiff bases and their metal complexes represent a very important class of bioactive coordination complexes because of their extreme structural diversity and high coordinating ability. They readily chelate an enormous variety of metal ions to give stable complexes with a broad variety of physicochemical and electronic properties, which are the foundation for their immense array of biological activities and are the reasons they are valuable potential therapeutic and drug candidates. Significantly, a number of studies have revealed Schiff base metal complexes to possess significant antibacterial activity against both Gram-positive and Gram-negative bacteria, a bi-spectrum activity that increases their potential as therapeutic agents for the treatment of infections due to a broad variety of microbial pathogens. Besides antibacterial activity, pharmacological interest is further extended to a number of therapeutic areas, including antifungal, antiviral, anticancer, and anti-inflammatory activities. Their bioactivity diversity underscores their potential as lead structures for drug development and discovery, particularly in the search for novel agents to counter the menace of drug resistance [3].

Schiff bases are usually obtained by a condensation reaction in which a primary amine makes nucleophilic attack on the electrophilic carbonyl carbon atom of an aldehyde or ketone. In this reaction, a molecule of water is eliminated and is replaced by an azomethine (C=N) group in place of the carbonyl (C=O) function [4]. The reaction is normally simple, typically under mild conditions and with minimal purification procedures, and this has served to render Schiff bases so widely favored within synthetic chemistry. Their structural makeup can be readily modified by altering the amine or carbonyl precursor, hence enabling great diversity in the nature of ligands that can be designed with specifically tailored steric and electronic properties. This flexibility is supplemented by their effective donor potency through the imine nitrogen, such that Schiff bases can coordinate effectively and efficiently with a vast range of metal ions under rapid conditions to form thermodynamically stable chelates. Such synthon versatility, molecular diversity, and affinity for complexation has made them essential ligands in coordination chemistry, with some examples of use branching to catalysis, materials science, and bioinorganic research [5]. Biologically, Schiff-base derivatives such as pyrimidines, imidazoles, hydrazines, and hydrazides have demonstrated significant antibacterial, anticancer, antifungal, antiproliferative, antipyretic, and antiviral properties [6, 7].

Hydrazine derivatives are of particular interest due to their wide range of pharmacological activities, such as anticancer [7, 8], anti-inflammatory, antifungal, antiplatelet [9], and antibacterial activities [10]. The presence of hydrogen-bond acceptors (C=O) and donors (–NH–NH₂) in these derivatives allows for supramolecular interactions that are liable for stabilizing and extending the structural framework of their metal complexes [11, 12]. Novel hydrazone derivatives (2a–g) were synthesized and exhibited potent multitarget inhibition of AChE, BChE, hCA I, and hCA II, surpassing standard drugs in some cases. Molecular docking and ADME predictions supported their strong binding affinities and favorable drug-like properties. Zinc, one of the metals frequently used to form complexes with these ligands, is an essential element in biological systems. It functions as an immunoregulatory agent with well-established anti-apoptotic and anti-inflammatory properties [13]. Benzotriazole-1-carbaldehyde, also a key aspect of ligand design, finds extensive industrial application—most notably as an extremely effective inhibitor of copper corrosion. Its adsorption behavior on Cu(110) surfaces has been investigated in great detail by the use of scanning tunneling microscopy, X-ray photoelectron spectroscopy, high-resolution electron energy loss spectroscopy, and theoretical solid-state calculations [14, 15]. In addition, hydrazide–hydrazone functional groups are now extremely promising pharmacophores in modern rational drug design because they exhibit multifarious biological activity with special relevance to their tremendous anticancer potential [14, 15]. The functional groups commonly interact selectively with biomolecular targets to enable the modulation of critical biochemical pathways of disease pathogenesis. Their spatial flexibility and availability of multiple donor atoms enhance their affinity for both enzymes and metal ions, thereby widening their therapeutic potential. In silico investigations have increasingly validated the biological activity of Schiff-base ligands and their metal complexes. Computational strategies—principally molecular docking—have been applied to model their binding with putative protein targets (e.g., PDB IDs 1GAL, 2AZ5, and 1HNJ), generating quantitative binding energies in addition to detailed hydrogen-bonding network topology maps and hydrophobic contacts that are fed into structure–activity relationship (SAR) analyses. Supporting DFT calculations also clarify electronic structure, frontier molecular orbitals, and reactivity descriptors. Coupling these computational tools with experimental data therefore creates a powerful platform for rationalizing bioactivity and guiding optimization of Schiff-base–derived drug leads [16, 17].

This work reports the synthesis of a new heterocyclic Schiff base ligand, 2,2′-((4-chloro-1,3-phenylene)bis(oxy))bis(N′-((E)-(1H-benzo[1-3]triazol-1-yl)methylene)acetohydrazide), and coordination with cobalt(II) and zinc(II) chlorides to give two different metal complexes. The compounds were completely characterized by spectroscopy and tested for antibacterial activity. A complete density functional theory (DFT) was conducted to unveil their electronic structures, bonding properties, and reactivity parameters useful for their biological modes of action.

## Experimental

### Physical measurements

All reagents and chemicals used in this study were purchased from commercial sources and employed as they were received without further purification. The synthesized ligand (L) was structurally characterized by proton (¹H) and carbon-13 (¹³C) nuclear magnetic resonance, which was operated in DMSO-d6 against the internal standard tetramethylsilane (TMS) using a Bruker Avance spectrometer (500 MHz for ¹H, 125 MHz for ¹³C). Functional groups were assessed by Fourier-transform infrared (FT-IR) spectroscopy: spectra were recorded in the 4000–200 cm⁻¹ range using a Shimadzu FT-IR 8400 S and additionally in the 4000–400 cm⁻¹ range using a Biotic 600 FT-IR; samples were absorbed as both CsI and KBr pellets to maximize resolution. Electronic absorption spectra of the ligand and metal complexes were recorded by UV–Vis spectrophotometry (Shimadzu UV-Vis 160-A) in 10⁻³ M DMSO solution with a 1.0 cm quartz cuvette. Molecular ion and fragmentation details for the ligand were obtained by mass spectrometry (ESI-MS) on a Sciex instrument.

Conductivity titrations of metal complexes were obtained at 25 °C in 10⁻³ M DMSO on a calibrated Eutech Cyberscan 510 digital conductivity meter. The magnetic susceptibilities used in deducing electronic structures were obtained at room temperature using Sherwood Scientific apparatus. Elemental (C, H, N) analyses were performed on an Ager 300 instrument equipped with an EA1112 analyzer. Metallic content was ascertained with atomic absorption spectrophotometry (Shimadzu A.A-7000), and chloride content was determined with potentiometric titration on Metrohm 686-G titration equipment. Thermal properties were then tested with a Stuart SMP40 digital melting point instrument to evaluate for reproducibility.

### Materials

The chemical reagents used in this study were ethyl chloroacetate, benzotriazole-1-carbaldehyde, potassium carbonate, hydrazine hydrate (80%), potassium hydroxide, ethanol, acetone, methanol, 4-chlorobenzene-1,3-diol, glacial acetic acid, dimethylformamide (DMF), and dimethyl sulfoxide (DMSO). These materials were all ordered from established providers (Sigma-Aldrich and Scharlau) to ensure a consistent quality with the ability for synthetic and analytical process aptitudes. Reagents were stored according to the manufacturers’ recommendation and were used without purification.

### Synthesis of the precursor {2,2′-((4-Chloro-1,3-phenylene)-bis(oxy))-di(acetohydrazide)}[P]

To According to a normal procedure, 4-chlorobenzene-1,3-diol (9.0 g, 62.2 mmol) was dissolved in 30 mL of acetone, to which ethyl chloroacetate (17.0 g, 138.7 mmol) and potassium carbonate (8.7 g, 62.5 mmol) were added. The reaction mixture was refluxed over a water bath; after reflux for a total period of 48 h, the reaction gave diethyl-2,2′-((4-chloro-1,3-phenylene)bis(oxy))diacetate as a clear brown oil (8.8 g, 47% yield). This diester was suspended in 25 mL of ethanol and reacted with hydrazine hydrate (2.9 g, 28.9 mmol) to give 2,2′-((4-chloro-1,3-phenylene)bis(oxy))di(acetohydrazide)[P]. The reaction mixture was refluxed for an additional 3 h, filtered, and washed several times with hot diethyl ether and ethanol, giving a white solid (3.4 g) that melts and decomposes at 227–229 °C.

Characterization by FT-IR (KBr) revealed bands at ν(N–H₂) 3296–3322 cm^−^¹, ν(N–H) 3192 cm^−^¹, ν(C=O) 1674 cm^−^¹, and ν(C–O) 1362 cm⁻¹ [16]. The elemental analysis for carbon (C), hydrogen (H), nitrogen (N), and chlorine (Cl) calculated the following percentages: C 41.61%, H 4.54%, N 19.41%, Cl 12.28%; while the found percentages were: C 41.57%, H 4.51%, N 19.39%, Cl 12.24%.

### Synthesis of ligand

A solution of the precursor P (1.70 g, 6.33 mmol) was prepared in 25 mL of methanol. Benzotriazole-1-carbaldehyde (2.17 g, 12.6 mmol) was added portion wise in three installments with the assistance of glacial acetic acid, using an additional 10 mL of methanol as catalyst/solvent. The reaction mixture was refluxed for 3 h. Upon completion, the crude material was recrystallized from methanol and then dried with diethyl ether to provide the ligand as a white solid (2.3 g, 61% yield); mp 265–270 °C.

### Synthesis of Co and Zn complexes

The metal complexes were prepared similarly. In a typical synthesis, L (0.20 g, 0.30 mmol) was dissolved in ethanol (10 mL) with 4–5 drops of DMF and then stirred with a solution of the respective metal chloride (MCl_2_; 0.16 g–0.13 g, respectively) in ethanol (10 mL). The mixture was refluxed for 3 h in a deliberate attempt to bring about a complete reaction. When cooled to room temperature, a brown precipitate formed slowly; the solid was washed successively with 5 mL portions of ethanol and diethyl ether to clean it, filtered, and recrystallized from ethanol. The product was vacuum-dried. For example, [Co(L)]Cl_2_ was obtained as a brown solid (0.16 g, 57% yield; mp > 300 °C). Table 1 displays the physical data and reagent amounts for all the complexes.

### Synthesis of ZnO and Co_3_O_4_ NPs

Synthetic Zn(II) and Co(II) Schiff-base complexes were thermally degraded at 600 °C for 5 h under a controlled atmosphere. The annealing at high temperature caused complete decomposition of the organic ligand structure and yielded zinc oxide (ZnO) and cobalt oxide (Co_3_O_4_) NPs, respectively.

### Theoretical approach

All of the quantum-chemical calculations were achieved with the Gaussian 09 program [18]. Gas-phase structures of the free ligand (L) and its metal complexes, [Zn(L)]Cl₂ (1) and [Co(L)]Cl₂ (2), were fully optimized by density functional theory (DFT) with the hybrid B3LYP functional [19]. For the non-metal atoms (C, H, N, O, and Cl), the 6-31G+(d, p) basis set—incorporating polarization and diffuse functions—was employed, and the transition-metal centers (Zn(II) and Co(II)) were represented by the LANL2DZ effective core potential and basis set to incorporate core electrons and relativistic effects [20]. This was to strike a balance between computational efficiency and accuracy, in accordance with previous studies on analogous coordination compounds.

The geometry optimizations were converged to Gaussian’s default tight criteria (maximum force ≤ 0.00045 Hartree/Bohr; maximum displacement ≤ 0.0018 Å). Harmonic vibrational frequency calculations at the same level confirmed the nature of the stationary points: the lack of imaginary frequencies pointed to true minima on the potential energy surface. The structures were then utilized to compute electronic and reactivity descriptors like frontier molecular orbital energies (HOMO and LUMO) and the HOMO–LUMO gap (ΔE_gap_), and global reactivity parameters such as chemical hardness (η), softness (σ), chemical potential (µ), electronegativity (χ), and electrophilicity index (ω). Molecular electrostatic potential (MESP) maps were also calculated to visualize the charge distribution and identify regions susceptible to nucleophilic or electrophilic attack.

## Results and discussion

The Physical and analytical information for the prepared compounds is tabulated in Table 1. The compounds were soluble in DMSO and exhibited satisfactory ambient stability: upon storage in sealed containers at room temperature, they maintained color and exhibited no sign of precipitation or decomposition over several weeks. Molar conductance measurements are in accordance with an electrolytic character, and qualitative testing using AgNO_3_ indicated that chloride anions are found outside the coordination sphere.

**Table 1:** Physical properties of the ligand and its complexes

Compounds	M.wt	Calculated (%) found			Metal%	_M_Ω^−1^cm^2^mol^−1^
		C	H	*N*		
C _24_ H_19_ ClN _10_O _4_	546.93	52.65 52.63	3.47 3.44	25.59 25.53	**–**	**–**
C_24_H_19_CoCl_3_N_10_O_4_	676.76	42.55 42.48	2.81 2.77	20.68 20.62	8.71 8.69	69.5
_24_H_19_ ZnCl_3_N_10_O_4_	683.21	42.15 42.11	2.78 2.73	20.49 20.45	9.57 9.55	72.6

### FTIR and NMR spectra

IR data are listed in Table 2. The free ligand exhibits characteristic peaks at 3371 cm^−1^ ν(N–H) and 1649 cm^−1^ [21]. ν(C=N) (imine) is 1620 cm^−1^ [22]. For the metal complexes, the carbonyl (amide) stretch is 1664–1622 cm^−1^ and at a lower frequency relative to the free ligand [23], consistent with delocalization of electron density through (dπ–pπ interaction) back-bonding on coordination. The ν(C=N) stretches (1590–1614 cm^−^¹) also shift to lower frequency on complex formation, favoring imine-nitrogen coordination to the metal center [24]. Further bands observed at 430–470 cm^−^¹ are attributed to ν(M–N) vibrations, also in favor of coordination through the imine nitrogen [21]. These spectral changes confirm previous information and favor the proposed coordination mode and structural assignments [23]. Complementary molar conductance measurements performed in dimethyl sulfoxide (DMSO) further support these conclusions, revealing values characteristic of a 1:2 electrolyte type. This kind of behavior suggests that the complexes are split up in solution to leave two counter ions per formula unit, the type of behavior ordinarily described by high ionic mobility and willingness to undergo great electrochemical activity. These combined spectroscopic and physicochemical findings provide consistent support for the proposed structures and their electrolyte nature and thus for both the synthetic method and molecular design envisioned.

**Table 2:** IR spectroscopy information of the ligand and its complexes

Compounds	ν (NH)	ν (C=O)	ν (C=*N*)	ν (M -*N*)
[L]	3371	1649	1620	–
[Co(L)]Cl_2_	3304	1664	1608	430
[Zn(L)]Cl_2_	3361	1622	1589	470

**Fig. 1:**
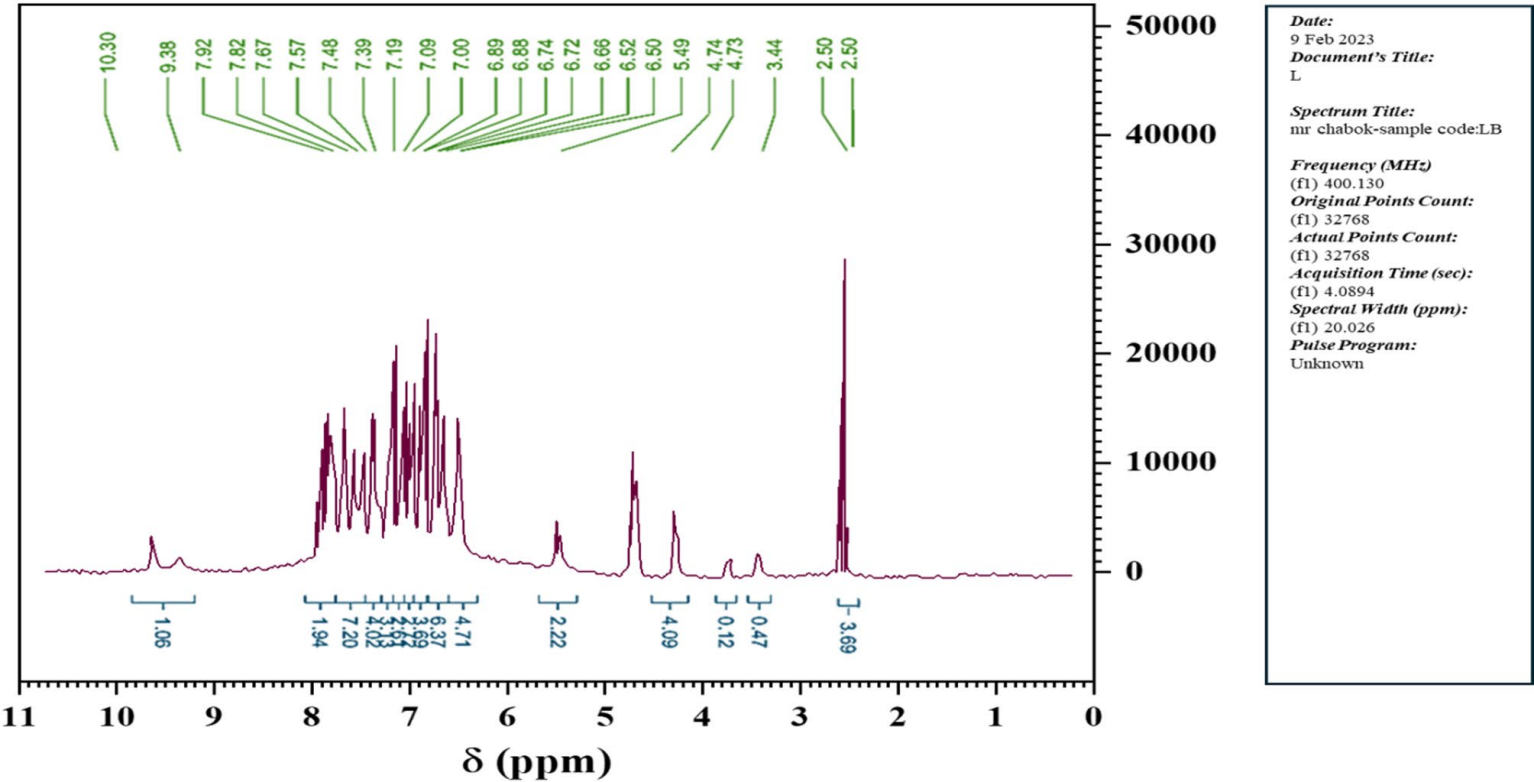
^1^H-NMR spectrum in (DMSO-d_6_) for the L ligand

 ¹H NMR spectrum of the ligand (Fig. 1) show ^1^H-NMR spectrum in (DMSO-d6) for the L ligand the presence of signals both in the aliphatic and aromatic regions [25]. Aromatic resonances at 7.92–6.52 ppm arise due to aromatic protons. A singlet at 4.74 ppm (4 H, s, CH₂) arises due to tautomerism of the Schiff-base unit and corresponds to CH₂ protons at C(9,9′)–H. Resonances at 6.52 ppm (s, 1H) and 6.59 ppm (s, 1H) correspond to C(15) and C(13), respectively. The singlet at 7.38 ppm (2H, s, CH_3_) corresponds to C(12′)–H, while the doublets at 7.40–7.41 ppm (4 H, d, J_HH_ = 5 Hz) and at 7.92–7.91 ppm (2 H, d, J_HH_ = 5 Hz) correspond to aromatic protons C(3,3′,4,4′) and C(2,2′,5,5′′), respectively [26, 27]. Azomethine protons (N=C–H, C7,7′)-H appear as a sharp singlet at 9.45 ppm (s, 2 H) [26], while N–H protons appear at 10.30 ppm (2H, s).

**Fig. 2:**
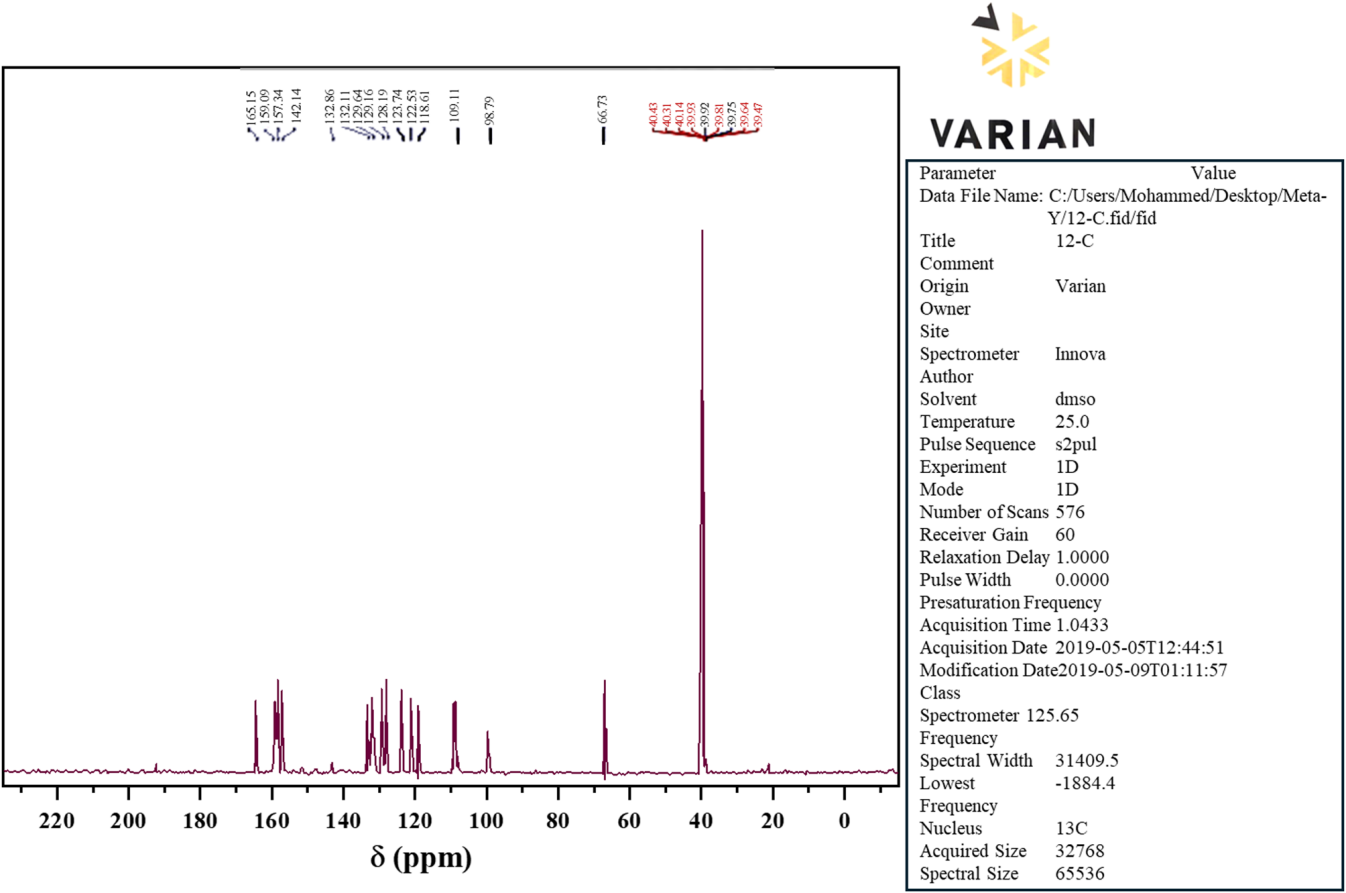
^13^C-NMR spectrum in (DMSO-d_6_) of L ligand

The ¹³C NMR spectrum (Fig. 2) exhibits ^13^C-NMR spectrum in (DMSO-d^6^) of ligand resonances in conformity with the assigned carbon environments. A resonance at 66.73 ppm corresponds to C(9,9′), whereas C(15) resonates at 98.79 ppm. The aromatic carbons correspond to the following multiple signals at 109.11, 118.61, 122.53, 123.74, 128.19, 128.61, 129.16, 132.11, 132.86, 157.34, and 158.09 ppm, specifically C(13,5,5′,11,2,2′,3,3′,4,4′,6,6′,1,1′,12,10, and 14). The maximum at 142.74 ppm is attributed to C(7,7′) (H–C=N), and an appearance at ≈ 165.15 ppm is assigned to a conjugated carbonyl (C=O) at C(8,8′), indicating imine tautomerization to a carbonyl function [26]. The emergence of this carbonyl resonance verifies the presence of tautomerism, increased electron delocalization, and potential intramolecular hydrogen bonding factors that may affect the donor character of the ligand and consequently the geometry, stability, and reactivity of the resulting complexes.

**Fig. 3:**
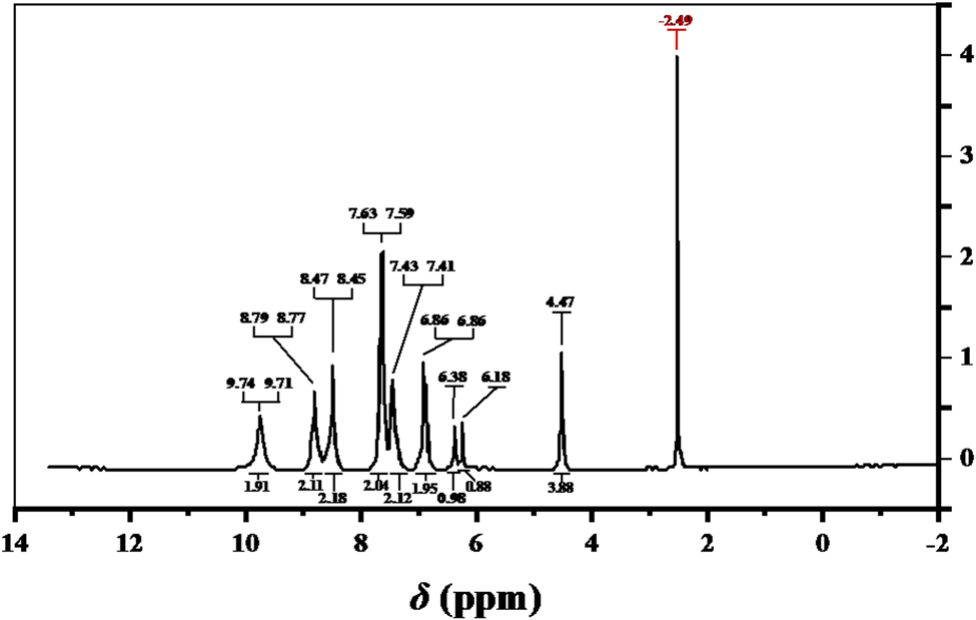
^1^H-NMR spectrum in DMSO-d6 for the [Zn(L)]Cl_2_ complex

The ¹H NMR spectrum of [Zn(L)]Cl_2_ (Fig. 3) show ^1^H-NMR spectrum in DMSO-d^6^ for the [Zn(L)]Cl_2_ complex signals for the ligand backbone with modest chemical-shift differences upon coordination. In the aliphatic region, CH₂ protons (C(9,9′)–H) appear as a singlet at δ = 4.47 ppm (4H, s, CH₂). Aromatic resonances are characterized by a singlet at δ = 6.18 ppm (1H, s, C(15)–H), a doublet at δ = 7.43–7.41 ppm (2 H, d, J_HH_ = 10 Hz, C(3,3′,4,4′)–H), a singlet at δ = 6.86 ppm (2 H, s, C(12)–H), and a doublet at δ = 7.59–7.63 ppm (2 H, d, J_HH_ = 20.00 Hz, C(2,2′,5,5′)–H). Other δ = 6.38 ppm (2 H, s, C(13)–H) and δ = 8.47 ppm (2 H, s, azomethine N=C–H, C7,7′) signals occur. The N–H resonances shift downfield to δ = 9.74 ppm (2 H, s) as would be expected for deshielding on coordination (roughly a 2 ppm downfield shift from the free ligand). Residual DMSO-d6 and traces of water in the solvent are responsible for signals at δ = 2.49 ppm and δ = 3.34 ppm, respectively.

### Mass spectra

**Fig. 4:**
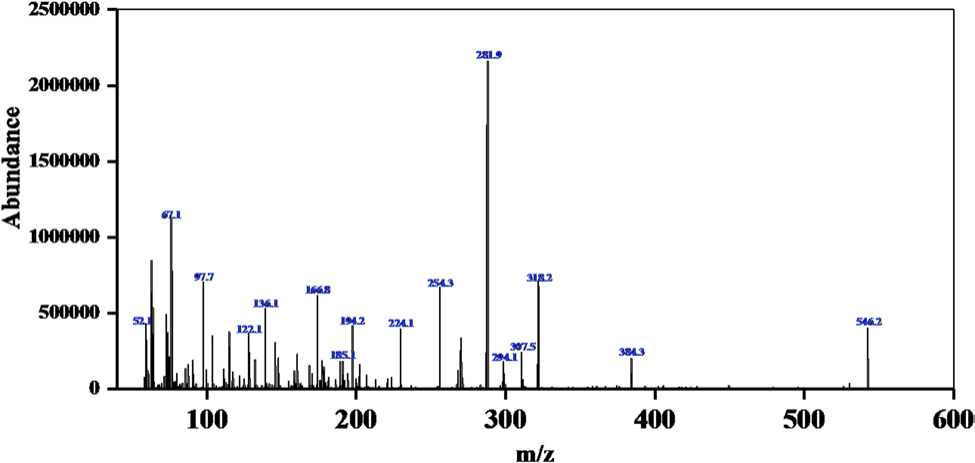
The electrospray (+) mass spectrum of ligand L

The mass spectrum of the ligand shows a peak corresponding to its protonated molecular ion, [C_24_H_19_ClN_10_O_4_ + H]^+^ at m/z = 546.13 amu as shown in (Fig. 4) show the electrospray (+) mass spectrum of ligand L. These results support the proposed structural formulas for the prepared ligand.

### Electronic spectra and magnetic moment measurements

The free ligand (L), in DMSO, exhibits three strong UV–visible absorption bands at 265, 321, and 358 nm. The energetic band at 265 nm corresponds to π→π* transitions of the conjugated aromatic system. The 321 and 358 nm bands are attributed to n→π* transitions, arising from promotion of heteroatom non-bonding electrons (predominantly at N or O) to antibonding π* orbitals corresponding to the azomethine and other conjugated parts. These bands reflect the electronic organization of the ligand, e.g., its degree of conjugation and the effect of electron-donating or withdrawing substituents. The appearance of distinct π→π* and n→π* bands together is a characteristic feature of a highly delocalized system and can make a significant impact on the optical properties of the ligand and coordination chemistry with metal ions [28, 29].

There are intra-ligand bands in the range 266–276 nm in the complexes, and the other absorptions between 323 and 436 nm correspond to charge-transfer (CT) transitions. The 602 and 645 nm bands in the electronic spectrum of [Co(L)]Cl_2_ are attributed to the ^4^A_2_→^4^T_1_ d–d transition characteristic of a tetrahedral coordination sphere for the Co²⁺ion. Magnetic moment values also establish a tetrahedral geometry for the cobalt complex [30]. UV–Vis spectral features and magnetic data for the compounds in question are depicted in Table 3.

**Table 3:** Electronic spectra data and magnetic moment of Co and Zn complexes

Compounds	λ (nm)	Assignment	Suggested geometry	µ BM
[Co(L)]Cl_2_	333 436 602 645	C.T C.T ^4^A_2_→^4^T_1_^(p)^ ^4^A_2_^(F)^ → ^4^T_1_	Tetrahedral	5.16
[Zn(L)]Cl_2_	365	C.T	Tetrahedral	Dia

### TEM images and X-ray diffraction

**Fig. 5:**
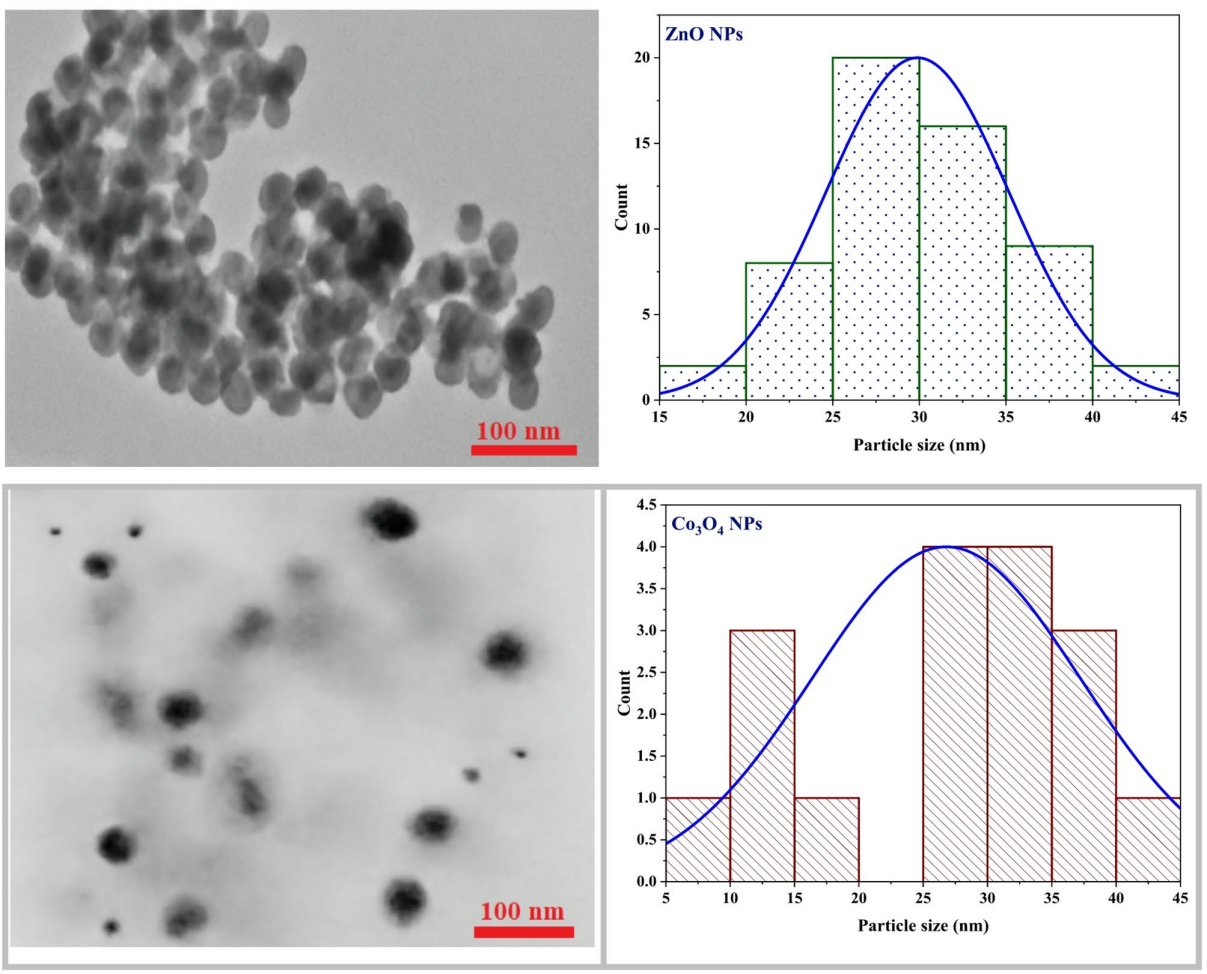
The TEM images and their corresponding particle size distributions of ZnO NPs and Co_3_O_4_ NPs

Transmission electron microscopy (TEM) was used to identify the particle-size distribution and structure of the metal-oxide NPs synthesized. Figure 5 displays the TEM image of ZnO NPs which exhibits a predominantly spherical morphology with a narrow size distribution. Statistical analysis of 57 particles reveals an average particle size of approximately 30 nm, with sizes ranging from 15 to 45 nm. The uniformity and limited agglomeration observed in the image indicate a well-controlled synthesis process, making the ZnO NPs promising candidates for coordination with Schiff base ligands.

**Fig. 6:**
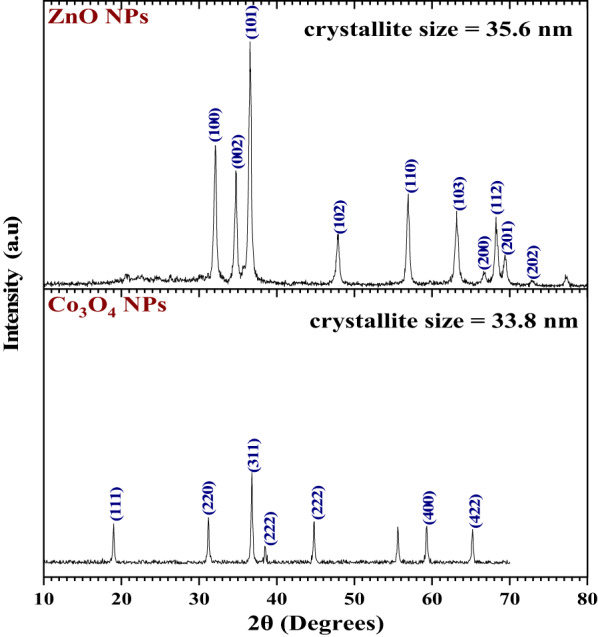
The XRD patterns of ZnO NPs and Co_3_O_4_ NPs

X-ray diffraction (XRD) profiles of ZnO and Co_3_O_4_ NPs (Fig. 6) confirm their crystalline nature and phase purity. The ZnO pattern corresponds to the hexagonal wurtzite structure (JCPDS no. 36–1451) with well-defined peaks at 2θ = 32.14° (100), 34.76° (002), 36.61° (101), 47.86° (102), 56.86° (110), and 63.14° (103) [31, 32]. Preferred orientation in that direction is indicated by the highest peak at 36.61° ((101) plane). The dimensions of the crystallites determined from the most intense reflections using the Debye–Scherrer formula (i.e., practically the (101), (002), and (100) peaks) result in an average diameter of 35.6 nm; the relatively low full width at half maximum (FWHM) values (e.g., 0.1968° for peaks at 32.14° and 34.76°) reflect a homogenous nanoscale constitution [33].

The Co_3_O_4_ morphology is cubic spinel phase morphology (JCPDS no. 42–1467) with reflections at 2θ = 18.99° (111), 31.20° (220), 36.80° (311), 44.80° (400), 55.60° (422), 59.30° (511), and 65.19° (440) [34, 35]. The (311) reflection with the largest intensity at 36.80° was used in estimations of crystallite size; Scherrer analysis across a series of peaks yields an average crystallite size of 33.8 nm. Widely broader Co_3_O_4_ peaks (e.g., FWHM ≈ 0.2362° for the (311) peak) indicate a slightly wider crystallite-size distribution compared with ZnO but still well-crystallized material. Overall, crystallite sizes obtained from XRD are in reasonable agreement with TEM observation, confirming successful synthesis of nanoscale metal oxides appropriate for subsequent coordination with the Schiff-base ligand as well as for potential catalytic or biological application.

### DFT calculations

**Fig. 7:**
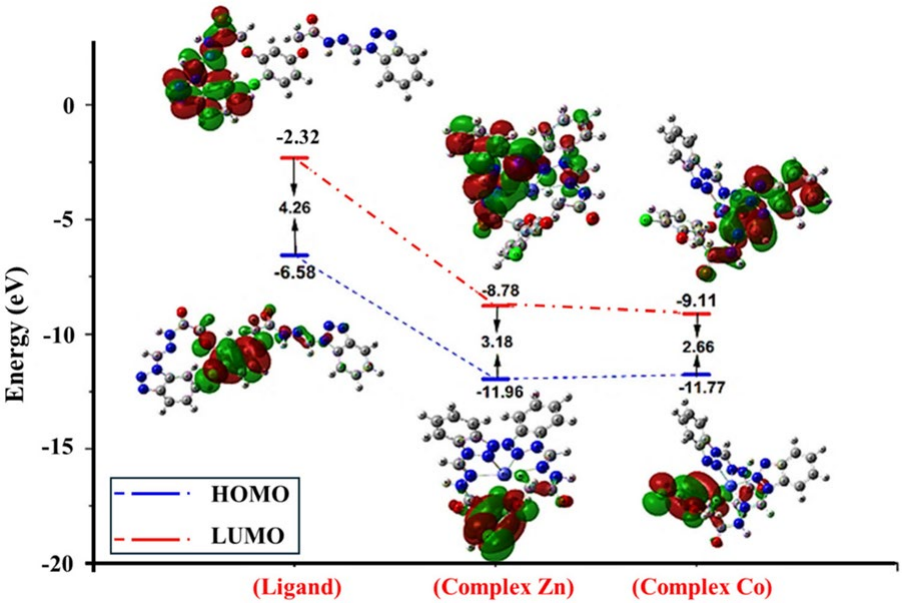
Optimized structures with HOMO and LUMO energy values for the [Zn(L)]Cl_2_ and [Co(L)] Cl_2_ complexes

Density Functional Theory (DFT) is a quantum chemical approach to predicting molecular properties energies, optimized geometries, vibrational frequencies, and reaction profiles based on the electron density as the variable of choice [36]. DFT calculations were employed in this work to optimize the geometry and perform vibrational analysis on the complexes in Fig. 7. All calculations were executed using Gaussian 16 [37]. Ligand atoms were described by the 6-31G + d, p basis set and the central metal ions by the Lanl2dz (Los Alamos National Laboratory double-zeta) basis set. Geometry optimizations were carried out with the hybrid B3LYP functional (Becke’s three-parameter exchange combined with the Lee–Yang–Parr correlation functional), a widely used functional that offers an outstanding balance between accuracy and computational cost. The Lanl2dz effective-core/basis set is appropriate for systems containing transition metals and is being routinely employed to obtain satisfactory results for heavy elements [38, 39].

#### DFT study of [M(L)] complexes where M=Zn and Co

The ground-state geometries of the [Zn(L)] and [Co(L)] complexes were optimized at the DFT/B3LYP level using Gaussian 16, and the structures were visualized using GaussView 6. Figure 7 presents the frontier molecular orbitals, which include the highest occupied molecular orbital (HOMO) and the lowest unoccupied molecular orbital (LUMO), along with their energy gap (ΔE_gap_). In the framework of molecular reactivity, the HOMO mainly depicts the electron-donating ability of a molecule, whereas the LUMO depicts its electron-accepting ability [40]. The HOMO–LUMO gap is therefore a measure of electronic stability and reactivity: a smaller ΔE_gap_ is generally related to greater polarizability, larger charge-transfer capacity, and greater tendency to chemical or biological activity [41].

In an attempt to establish additional quantification of electronic and chemical activity, several global reactivity descriptors have been developed from the frontier orbital energies. These are electronegativity (χ), electrophilicity index (ω), chemical hardness (η), softness (σ), and chemical potential (µ), as well as the isolated HOMO and LUMO energies and ΔE_gap_. The employed equations are listed in Table 4, and the corresponding calculated values are compiled in Table 5. These parameters, obtained within the framework of conceptual DFT, are consistent with Koopmans’ theorem for closed-shell molecular systems and provide a theoretical basis for understanding the observed chemical stability and reactivity trends [42, 43].

**Table 4:** Mathematical equations for calculating quantum chemical data in DFT

Quantum chemical descriptor	Equation
Energy gap $\:{\Delta\:}{E}_{\mathrm{g}\mathrm{a}\mathrm{p}}$	$\:{\Delta\:}{E}_{\mathrm{g}\mathrm{a}\mathrm{p}}=$ ${E}_{\mathrm{L}\mathrm{U}\mathrm{M}\mathrm{O}}-{E}_{\mathrm{H}\mathrm{O}\mathrm{M}\mathrm{O}}$
Chemical hardness (η)	$\:\eta\:=\frac{\left({\Delta\:}E\right)}{2}$
Softness (σ)	$\:\sigma\:=\frac{1}{\left(2\eta\:\right)}$
Electrophilicity index (ω)	$\:\omega\:=\frac{{\left(\mu\:\right)}^{2}}{2\eta\:}$
Electronegativity index (χ)	$\:{\upchi\:}=-\mu\:$

The negative values obtained for both EHOMO and ELUMO energies reflect the inherent electronic stability of the complexes, indicating a lower tendency to lose or gain electrons spontaneously. Nonetheless, when these negative values become significantly large, they can potentially weaken the coordination bonds by reducing the effective overlap between the metal center and the ligand orbitals, which may influence the overall complex stability. The parameters of chemical hardness (η) and softness (σ) are fundamentally interconnected and serve as crucial indicators of molecular reactivity and stability. Chemical hardness measures the resistance of a molecule to deformation or change in its electron cloud, while softness represents its ease of polarization and ability to participate in chemical reactions. Due to their inverse proportionality, an increase in hardness corresponds to a decrease in softness and vice versa [44]. These global reactivity descriptors play a pivotal role in confirming the proposed chemical structures, as well as in predicting their potential interaction sites and overall chemical behavior under various conditions.

The electrophilicity index (ω), as presented in Table 5, serves as an important parameter for assessing the electron-accepting capacity and overall reactivity of the studied molecules. Higher ω values typically correlate with stronger electrophilic behavior, indicating a greater tendency to attract electrons from donor species during chemical interactions. This electron transfer process often results in a decrease in the system’s total energy, stabilizing the resulting adducts or reaction intermediates [45]. Notably, the [Zn(L)] complex exhibits a relatively elevated electrophilicity index of 40.97 eV compared to the free ligand, suggesting enhanced biological potential and a more pronounced ability to function as an electron acceptor. Such characteristics imply a robust interaction between the ligand and the central metal ion, which may contribute to faster reaction kinetics and stronger coordination bonds [46]. Conversely, the chemical potential (*µ*) values for these complexes are negative, ranging from − 4.45 eV to −10.44 eV, indicating a thermodynamically favorable and stable electronic configuration that resists decomposition or spontaneous chemical change [47]. Among the metal complexes examined, the [Zn(L)]Cl_2_ species stands out with the lowest chemical potential, reflecting its superior stability relative to the others. Furthermore, the electronegativity (χ) values, all positive, denote the molecules’ inherent capacity to attract electrons, which aligns with their negative chemical potentials and suggests effective electron-withdrawing characteristics [48]. The [Co(L)]Cl_2_ complex demonstrates the highest electronegativity value at 10.44 eV, highlighting its strong affinity for electron density and potential implications in chemical reactivity and binding affinity.

Concerning the energy gap (Δ*E*_gap_) between the HOMO and the LUMO, the [Zn(L)]Cl_2_ complex exhibits the largest value, measuring 3.18 eV, in comparison to the [Co(L)]Cl_2_ complex. A wider energy gap typically correlates with decreased chemical reactivity and enhanced molecular stability, as it reflects a greater energy barrier for electron excitation from the HOMO to the LUMO level. This increased energy requirement implies that the [Zn(L)] Cl_2_ complex is less prone to participate in electron transfer processes, thereby making it more chemically inert under standard conditions. On the other hand, the free ligand displays an even larger energy gap of 4.26 eV, indicating an even higher level of stability and lower reactivity relative to its metal-bound counterparts. These variations in Δ*E*_gap_ values underscore the influence of metal coordination on the electronic properties of the complexes, suggesting that metal binding modulates the ligand’s reactivity and potentially its biological or catalytic activity [49, 50].

Concerning chemical hardness (η), the [Zn(L)]Cl_2_ complex is the highest amongst the compounds investigated, at 1.59 eV. In conceptual density functional theory, chemical hardness is an important descriptor of electronic stability of a molecule, or its resistivity to electron density changes due to external perturbation. Growing η value is related to an enhanced resistance of the molecule toward electronic structure deformation, indicating reduced polarizability and enhanced thermodynamic stability. Consistently, the greater hardness of the [Zn(L)]Cl_2_ complex suggests its more rigid electronic structure as compared to other studied species, which can influence its reactivity patterns, coordination chemistry, and potential in catalysis or biology. A higher hardness value signifies increased stability, as the molecule is less susceptible to electron transfer or perturbations in its electron cloud. This parameter is directly correlated with the HOMO–LUMO energy gap (ΔE), meaning that as ΔE increases, chemical hardness tends to rise as well, reinforcing the molecule’s inertness. Conversely, chemical hardness exhibits an inverse relationship with softness (σ), which quantifies the ease with which a molecule’s electron density can be distorted [49, 50]. These interrelated descriptors provide a comprehensive understanding of the electronic resilience and reactivity of the complexes, with the [Zn(L)] Cl_2_ complex demonstrating notable stability due to its elevated hardness value.

The calculated quantum chemical parameters provide a theoretical basis for the observed biological activities. The lower HOMO-LUMO energy gap of the [Co(L)] complex (2.66 eV) compared to the [Zn(L)] complex (3.18 eV) suggests its higher chemical reactivity. More importantly, the global electrophilicity index (ω) is markedly higher for both complexes ([Co(L)] Cl_2_ = 40.97 eV; [Zn(L)] Cl_2_ = 33.81 eV) relative to the free ligand (4.65 eV). This strong electrophilic character indicates that the complexes are potent electron acceptors, which may enhance their interaction with electron-rich biological macromolecules in bacteria, thus providing a rationale for their superior antibacterial performance [11].

**Table 5:** Determined energy levels corresponding to the HOMO and LUMO orbital energies represent the quantum physical properties of [Zn(L)] Cl_2_ and [Co(L)] Cl_2_ complexes

Compounds	EHOMO (eV)	ELUMO (eV)	ΔE_gap_ (eV)	η (eV)	σ (eV)	Dipole moment (debye)	µ (eV)	χ (eV)	ω (eV)	Total energy (kcal/mol)
Ligand	− 6.58	− 2.32	4.26	2.13	0.23	7.89	− 4.45	4.45	4.65	− 1402195.32
[Zn(L)]Cl_2_	− 11.96	− 8.78	3.18	1.59	0.31	10.85	− 10.37	10.37	33.81	− 1443030.50
[Co(L)]Cl_2_	− 11.77	− 9.11	2.66	1.33	0.37	11.36	− 10.44	10.44	40.97	− 1492895.56

### Molecular electrostatic potential (ESP) analysis

**Fig. 8:**
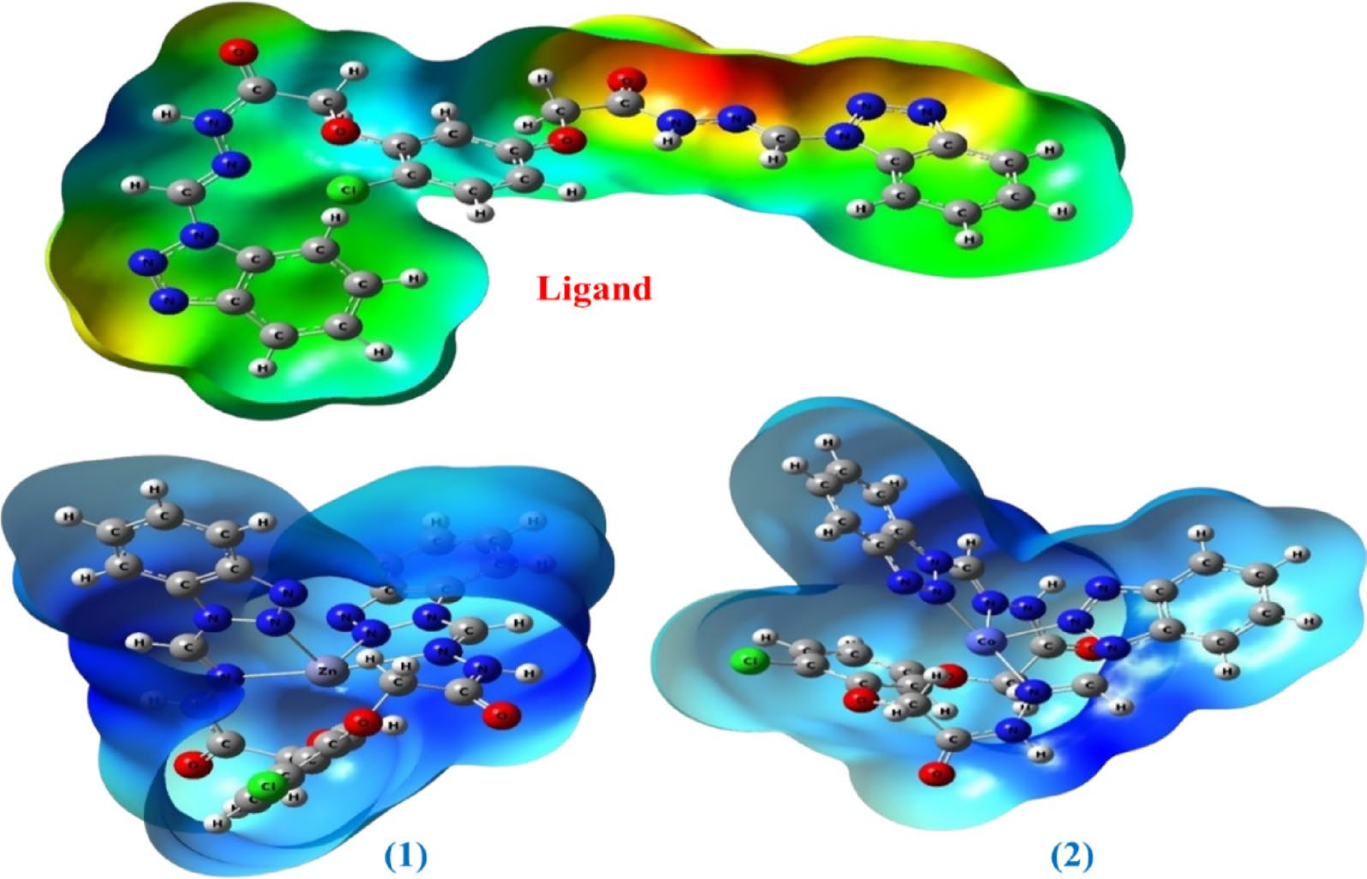
Electrostatic potential (ESP) of complexes of [Zn(L)] and [Co(L)]

Molecular Electrostatic Potential (MESP) analysis provides a map of the charge distribution on the surface of a molecule, allowing for the identification of potential sites for electrostatic interactions. MESP maps were calculated for the studied compounds (Fig. 8) to identify electron-rich regions (potential for electrophilic attack) and electron-deficient regions (potential for nucleophilic attack).

In the free ligand [L], regions with negative electrostatic potential (red areas) are primarily concentrated around the oxygen atoms of the carbonyl groups and the nitrogen atoms in the benzotriazole ring, making them potential sites for coordination with metal ions. Upon complexation, these regions become less negative, which confirms the occurrence of coordination. Regions with positive potential (blue areas) appear around the amide protons (N-H), indicating their potential involvement in hydrogen bonding. In the metal complexes, a positive region appears around the central metal ion, although it is surrounded by the negative electron density of the ligand. This charge distribution suggests that the complexes can interact with biological molecules through a variety of interactions, including direct coordination, electrostatic interactions, and hydrogen bonding. Understanding the MESP distribution can help interpret the potential mechanisms of the observed biological activity [51].

### Infrared (IR) spectroscopy

**Fig. 9:**
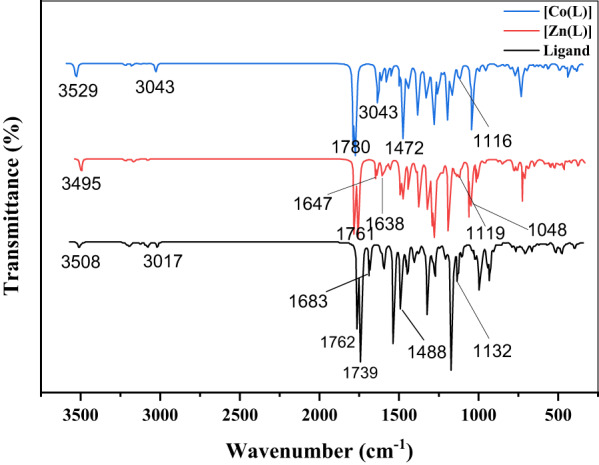
FTIR spectrum of the types [Zn(L)], [Co(L)] and Ligand

The IR spectrum of the complexes Zn(L) and Co(L), measured in the gas phase after optimization through theoretical studies (DFT), revealed characteristic electronic spectra. Figure 9 presents the FTIR spectra of the ligands and the complexes, with the relevant information extracted and summarized in Table 6.

**Table 6:** Comparative FT-IR frequencies (cm^−1^) of optimized ligand complexes: experimental vs. theoretical

Compounds	ν(NH)a/b (Exp)	ν (C = O) a/b (Exp)	ν (C = *N*) a/b (Exp)	ν (M -*N*) a/b (Exp)
**[L]**	3508/3381 (3371)	1739/1676 (1649)	1690/1629 (1620)	–
**[Co(L)]Cl**_**2**_	3529/3401 (3361)	1780/1715 (1622)	1629/1570 (1589)	470
**[Zn(L)]Cl**_**2**_	3495/3369 (3304)	1761/1697 (1664)	1638/1597 (1608)	430

^a^6-31G+(d, p) unscaling factor and

^b^6-31G+(d, p) with scaling factor 0.964

To enhance the agreement between the theoretically predicted and experimentally observed infrared (IR) spectra, a standardized frequency scaling factor was applied. This correction factor was sourced from the NIST Computational Chemistry Comparison and Benchmark Database (CCCBDB), which provides empirically derived adjustments designed to compensate for systematic overestimations in vibrational frequencies calculated by Density Functional Theory (DFT) methods. Implementing this scaling factor significantly improved the match between computed and experimental IR spectra, thereby confirming the reliability and accuracy of the computational model employed. Such an approach has been successfully utilized in prior studies involving nitroaromatic compounds and energetic materials, highlighting the vital role of the CCCBDB database in refining vibrational frequency assignments and enhancing the interpretative power of theoretical vibrational analyses.

The comparison reveals a generally good agreement between the experimental and theoretical vibrational frequencies, especially for the characteristic functional groups. Minor discrepancies are expected due to the inherent differences between gas-phase theoretical calculations and solid-state experimental measurements, as well as the anharmonicity of molecular vibrations not fully accounted for in harmonic frequency calculations. For instance, the theoretical N-H stretching frequencies are slightly higher than the experimental values, which could be attributed to intermolecular hydrogen bonding in the solid state that is not present in the gas-phase model. Similarly, the C = O and C = N stretching frequencies show reasonable correlation, with shifts upon complexation being consistent in both experimental and theoretical data, indicating successful coordination of the ligand to the metal ions. The appearance of new bands in the experimental spectra (e.g., ν(M-N)) confirms the formation of metal-nitrogen bonds, which are not explicitly listed in the theoretical table but are implicitly part of the optimized structures. This comparative analysis reinforces the structural assignments and validates the computational methodology employed in this study [52].

### Biological activity

**Fig. 10:**
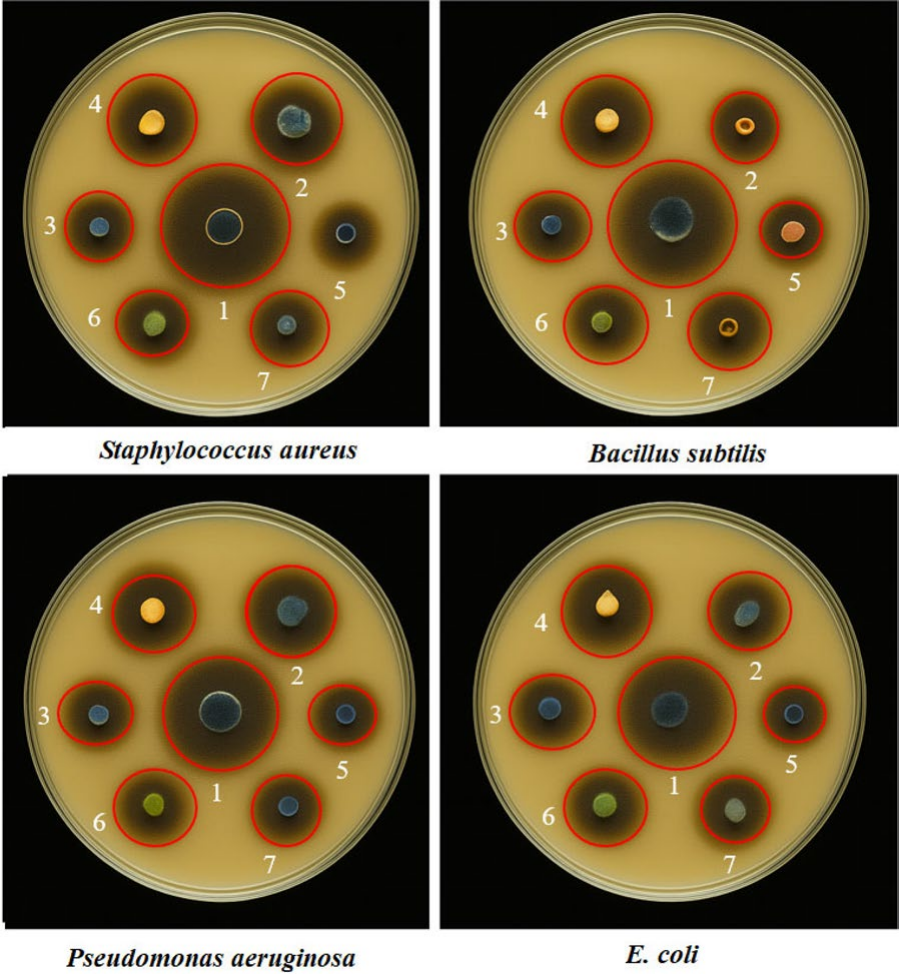
Inhibition diameter of ligands and their complexes

The biological activity of the ZnO NPs (1), Co_3_O_4_ NPs (2), [Zn(L)]Cl₂ (3), [Co(L)]Cl_2_ (4), ligand L (5), ZnCl₂ (6) and CoCl₂·6 H₂O (7), the biological efficacy of these compounds was evaluated against four bacterial strains: *Staphylococcus aureus*,* Bacillus subtilis*,* Pseudomonas aeruginosa*, and *Escherichia coli* (Fig. 10), the results showed clear differences in the diameters of inhibition zones, reflecting variability in the antibacterial efficacy of the tested compounds, Zinc oxide NPs and Co_3_O_4_ NPs exhibited the highest inhibitory activity, better than complexes, free ligands and metal salts. These results clearly demonstrate that antibacterial activity is influenced by the chemical structure and physicochemical characteristics of the compounds. The high efficacy of nanostructured metal oxides particularly ZnO is attributed to their ability to generate reactive oxygen species (ROS), which directly damage the bacterial cell wall and its essential components. A recent study reports that ZnO nanoparticles possess broad-spectrum antibacterial activity by inducing elevated ROS levels and causing membrane disruption, leading to bacterial cell death [52, 53]. Meanwhile, metal complexes benefit from the chelation effect, which increases lipophilicity and enhances membrane penetration [49, 53]. On the other hand, the absence of nanoscale features or chelating capacity in metal salts and the free ligand limits their inhibitory efficiency, as confirmed by the low values presented in Table 7. It is also noteworthy that Gram-negative bacterial strains (*E. coli* and *P. aeruginosa*) exhibited relatively higher resistance, with lower inhibition values compared to Gram-positive strains. This observation reflects the role of the complex outer membrane in limiting the effectiveness of antimicrobial agents except for ZnO, which maintained significant activity against these strains as well [50]. These findings are supported by a recent study conducted by Aziz, Shadha Nasser, et al. (2024), in which they synthesized and characterized a ternary nanocomposite consisting of ZnO and Co_3_O_4_ and evaluated its antibacterial activity against both Gram-positive and Gram-negative strains, including *S. aureus* and *E. coli*. The study revealed that the nanocomposite exhibited significantly enhanced antibacterial activity compared to the individual oxides, suggesting a synergistic effect resulting from the structural and physicochemical integration of the composite’s components. The authors attributed this elevated efficacy to the enhanced ROS generation capacity of the multi-oxide nanoparticles, along with improved penetration of bacterial cell membranes. These findings are consistent with the current study and further support the potential of metal oxides particularly ZnO and Co_3_O_4_ as a promising strategy for the development of potent, multi-target antimicrobial agents [54, 55].

**Table 7:** The biological activity of compounds

Compound codes	S. aureus	B. subtilis	*P*. aeruginosa	E. coli
1	28	27	26	24
2	24	22	21	18
3	19	18	17	17
4	17	17	16	16
5	13	12	11	11
6	9	10	8	9
7	10	9	7	8

## Conclusion

In this study, a novel Schiff base ligand and its Zn(II) and Co(II) complexes were successfully synthesized and comprehensively characterized through both experimental techniques and computational analyses. DFT calculations elucidated key electronic properties, revealing a decrease in the HOMO–LUMO energy gap (Δ*E*_gap_) from 4.26 eV in the free ligand to 3.18 eV and 2.66 eV for the [Zn(L)] and [Co(L)] complexes, respectively. This reduction indicates an enhancement in chemical reactivity upon complexation. Concurrently, the electrophilicity index (ω) exhibited a substantial increase from 4.65 eV in the ligand to 33.81 eV in [Zn(L)] and 40.97 eV in [Co(L)] suggesting a greater electron-accepting capacity, particularly notable in the Co(II) complex. The chemical hardness (η) also decreased progressively (ligand: 2.13 eV; [Zn(L)]: 1.59 eV; [Co(L)]: 1.33 eV), corroborating the increased reactivity trend. Electrostatic potential (ESP) mapping identified distinct nucleophilic and electrophilic regions localized around the coordination sites, consistent with the observed enhancement in antimicrobial activity. Biological evaluations conducted against a range of microorganisms including four Gram-positive bacteria such as *Bacillus subtilis* and *Staphylococcus aureus*, three Gram-negative bacteria including *Escherichia coli* and *Pseudomonas aeruginosa*, as well as two fungal species (*Candida* and *Trichomonas*) revealed that the metal complexes exhibited significantly greater antimicrobial activity than the uncoordinated free ligand.

## Supplementary Information

Supplementary Material 1.

## Data Availability

Data available for peer-reviewers under request.
