# Supplementary material for: Synthesis, characterization, DFT calculation, and biological activity of a new Schiff base ligand and its ZnO and Co3O4 nano-metal oxide complexes
Source: BMC Chem. 2025 Dec 12;20(1):10. doi: 10.1186/s13065-025-01673-1 (PMC12805709; doi:10.1186/s13065-025-01673-1)
Supplement: Supplementary file 1 — Supplementary Material 1. [file 13065_2025_1673_MOESM1_ESM.docx]

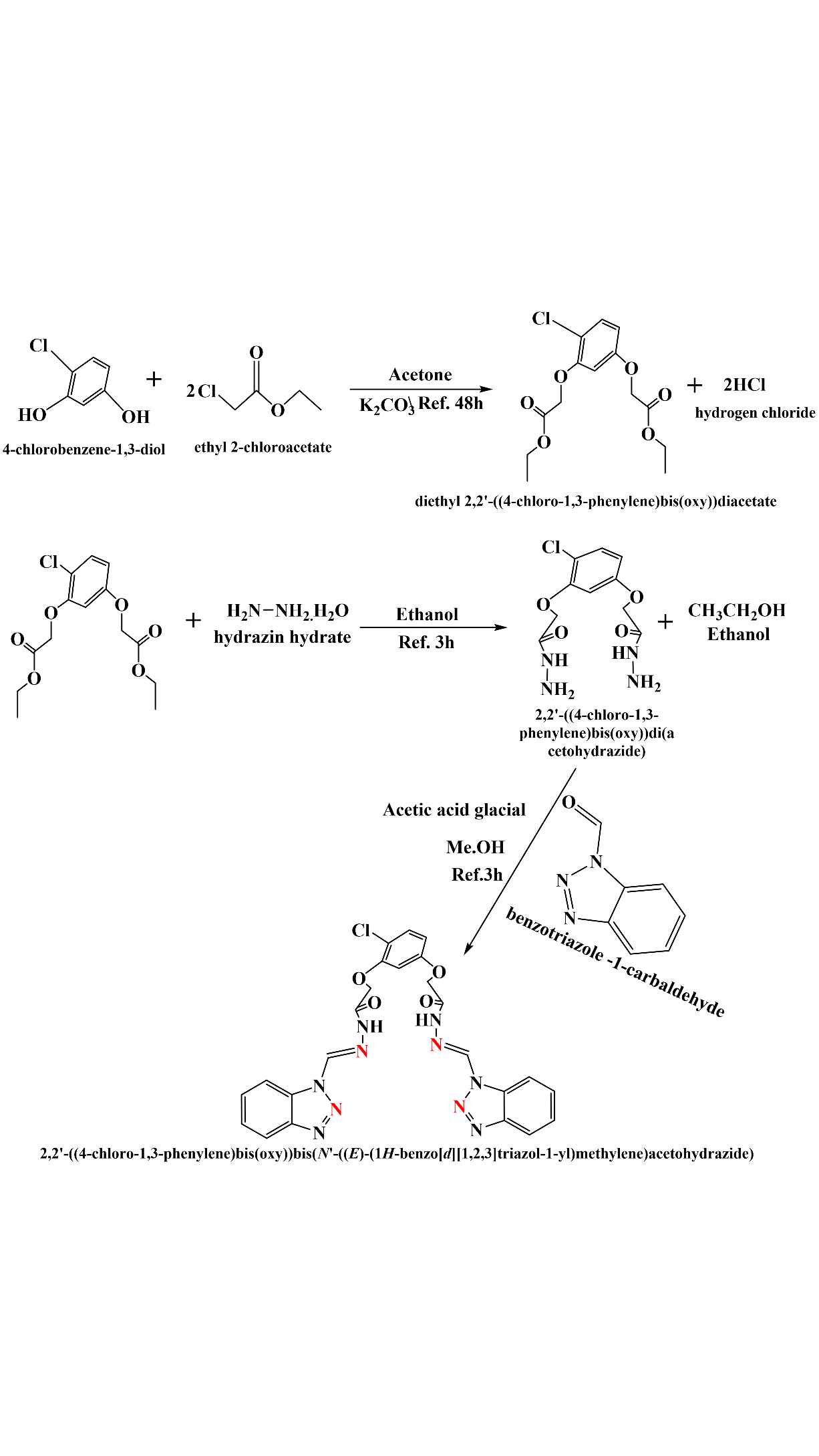


**Scheme 1:** Synthesis route of Schiff-base ligand [L]


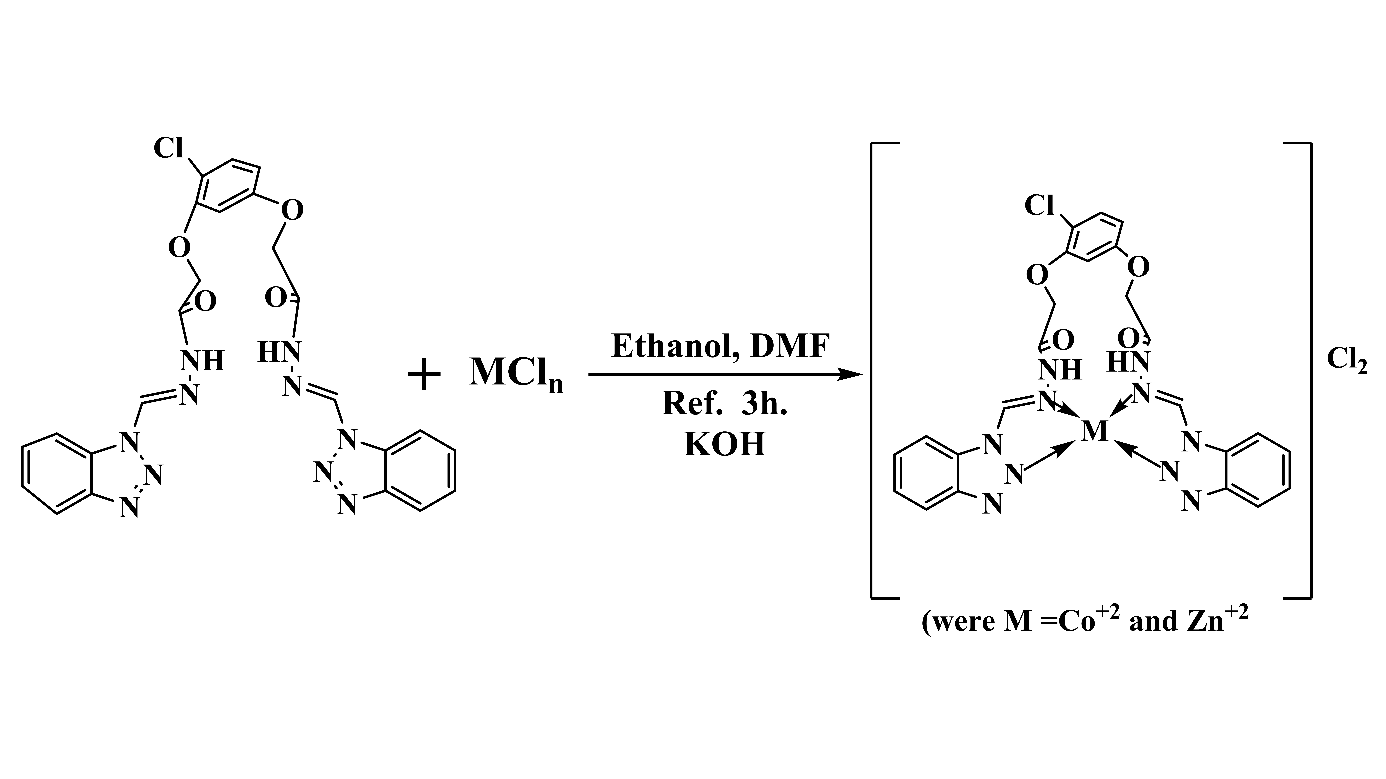


**Scheme 2.** General synthesis of complexes

**Table 2.** IR Spectroscopy information of the ligand and its complexes

| **Compounds** | **ν (NH)** | **ν (C=O)** | **ν (C=N)** | **ν (M -N)** |
| --- | --- | --- | --- | --- |
| **[L]** | 3371 | 1649 | 1620 | - |
| **[Co(L)]Cl_2_** | 3304 | 1664 | 1608 | 430 |
| **[Zn(L)]Cl_2_** | 3361 | 1622 | 1589 | 470 |


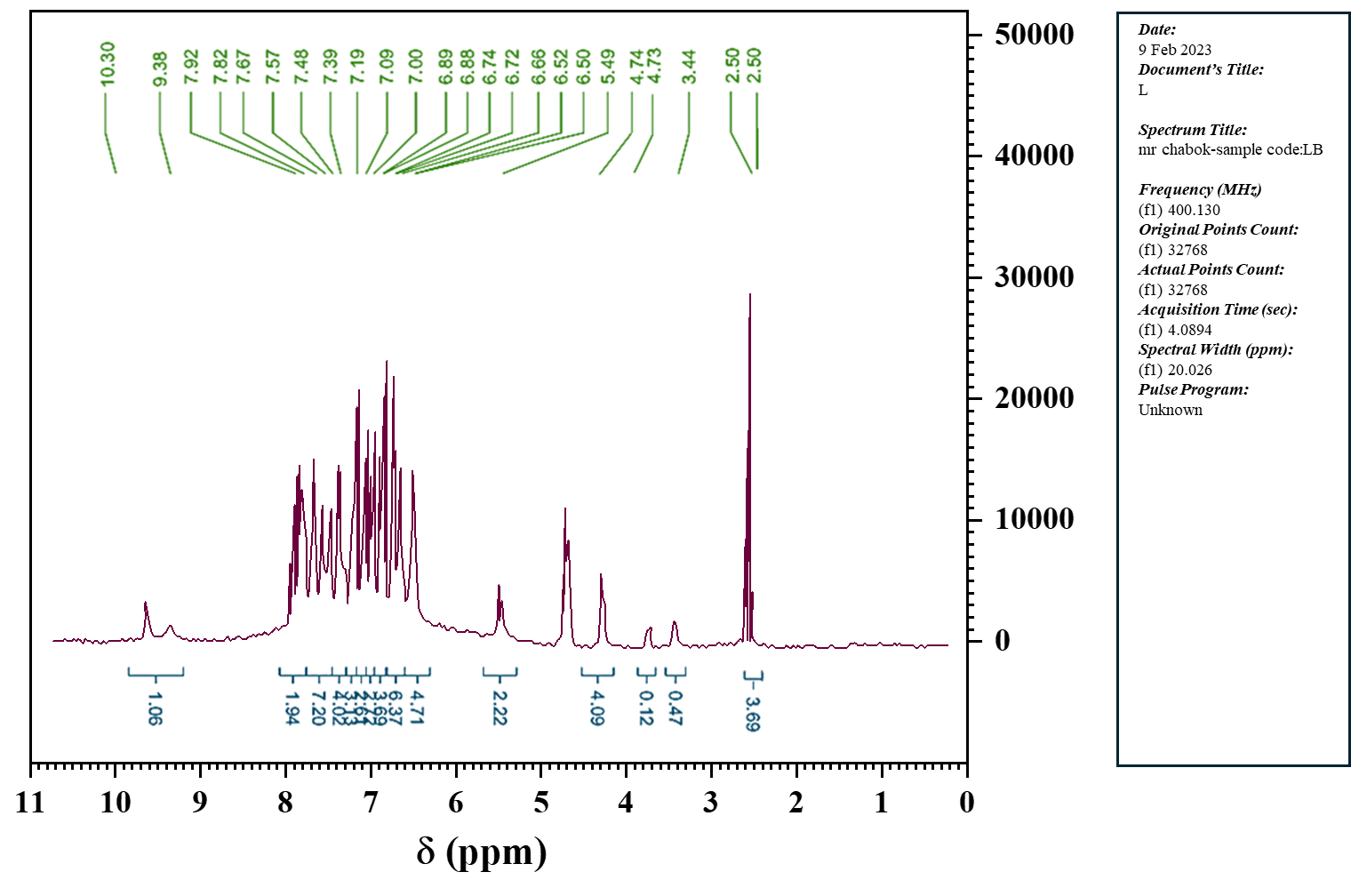


**Figure 3.** ^1^H- NMR spectrum in (DMSO-d_6_) for the L ligand


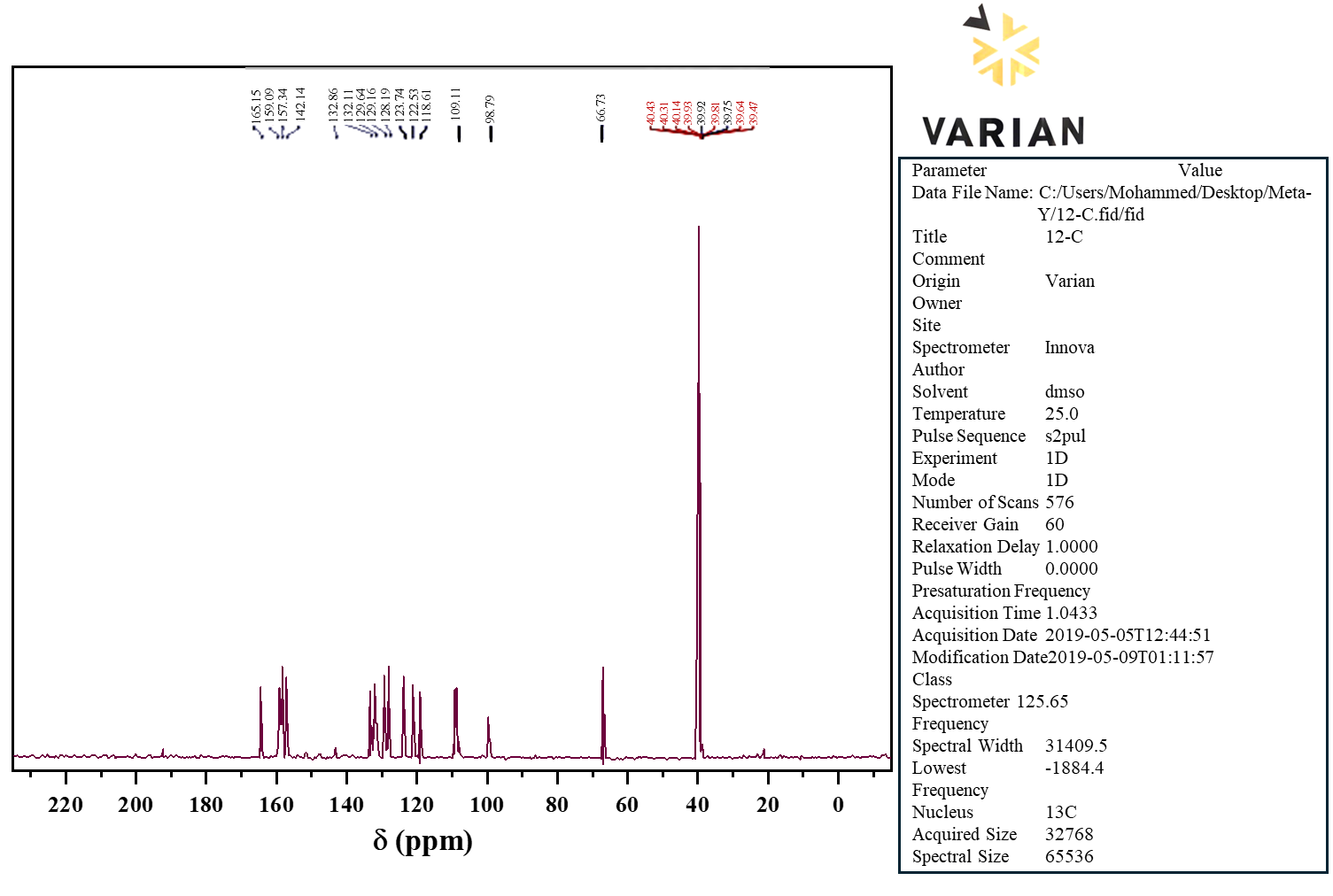


**Figure 4.** ^13^C-NMR spectrum in (DMSO-d_6_) of L ligand

**Figure 5.** ^1^H-NMR spectrum in DMSO-d6 for the [Zn(L)]Cl2 complex


**Figure 6.** The electrospray (+) mass spectrum of ligand L


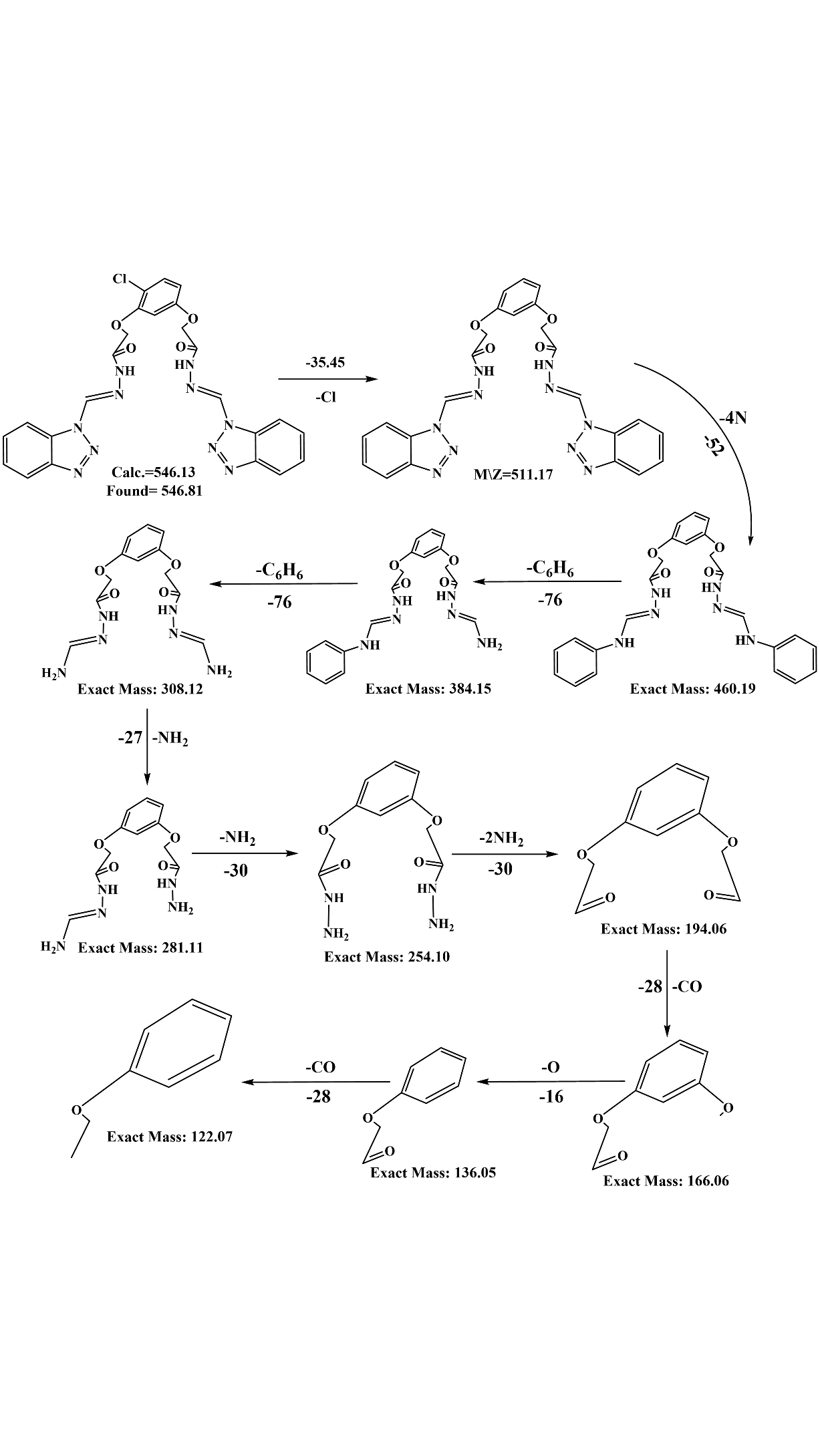


**Scheme 3.** The fragmentation pattern of ligand L
